# Deciphering the Role of the Gut Microbiota in Exposure to Emerging Contaminants and Diabetes: A Review

**DOI:** 10.3390/metabo14020108

**Published:** 2024-02-06

**Authors:** Xueqing Li, Huixia Niu, Zhengliang Huang, Man Zhang, Mingluan Xing, Zhijian Chen, Lizhi Wu, Peiwei Xu

**Affiliations:** 1 Zhejiang Provincial Center for Disease Control and Prevention, 3399 Bin Sheng Rd., Binjiang District, Hangzhou 310051, China; xqli@cdc.zj.cn (X.L.); 2111101061@nbu.edu.cn (H.N.); mlxing@cdc.zj.cn (M.X.); zhjchen@cdc.zj.cn (Z.C.); lzhwu@cdc.zj.cn (L.W.); 2 Disease Prevention and Control Center of Jingning She Autonomous County, Lishui 323500, China; hzljmx12688@163.com; 3 School of Public Health, Zhejiang Chinese Medical University, Hangzhou 310053, China; ggwszm15726626270@163.com

**Keywords:** emerging contaminants, emerging pollutants, gut microbiome, gut microbiota, glucose metabolism, diabetes

## Abstract

Emerging pollutants, a category of compounds currently not regulated or inadequately regulated by law, have recently become a focal point of research due to their potential toxic effects on human health. The gut microbiota plays a pivotal role in human health; it is particularly susceptible to disruption and alteration upon exposure to a range of toxic environmental chemicals, including emerging contaminants. The disturbance of the gut microbiome caused by environmental pollutants may represent a mechanism through which environmental chemicals exert their toxic effects, a mechanism that is garnering increasing attention. However, the discussion on the toxic link between emerging pollutants and glucose metabolism remains insufficiently explored. This review aims to establish a connection between emerging pollutants and glucose metabolism through the gut microbiota, delving into the toxic impacts of these pollutants on glucose metabolism and the potential role played by the gut microbiota.

## 1. Introduction

The gut microbiota, due to performing myriad vital functions within human health and being closely intertwined with human health and disease, has attracted increasing attention over the past decades. With deeper research into the gut microbiota, a broad range of functions have been recognized, encompassing carbohydrate digestion, the synthesis of vitamins and other nutrients, and the regulation of the immune system [1,2]. Given its crucial role in human health, the gut microbiota is considered a novel organ within the human body [3]. However, the structure and composition of the gut microbiota are highly susceptible to external compounds, leading to gut microbiota dysbiosis [4,5]. For example, exposure to 1–4 μm polystyrene microplastics for seven days led to significant differences between the microplastic-treated group and the control group in the Shannon and Simpson indices, with notable changes in the abundance of Bacteroidetes, Firmicutes, Proteobacteria, and Verrucomicrobia [6]. Alterations in the gut microbiota may impact host health through metabolic changes. The disruption of the gut microbiota caused by exogenous pollutants has been termed ‘gut microbiome toxicity’ [7]. Therefore, changes in the abundance and functionality of the gut microbiota following exposure to exogenous pollutants may represent a potential mechanism for pollutant-induced toxicity.

Emerging pollutants, defined as compounds that naturally occur or are synthetically produced and detectable and potentially harmful to the environment, flora, fauna, and humans, yet are currently not or inadequately regulated by law [6,8,9], have become a hot topic in toxicological research in recent years. Importantly, numerous studies have explored the link between emerging pollutants and the gut microbiota, demonstrating that exposure to emerging pollutants can impact the structure and functionality of the gut microbiota, thereby posing potential health risks to the host. Concurrently, the gut microbiota has a strong relationship with host metabolism, especially glucose metabolism. For example, Wang et al. studied the relationship between gestational diabetes mellitus and intestinal flora by recruiting pregnant women with gestational diabetes mellitus and studying the changes in their intestinal flora, and they found that the changes in certain intestinal bacteria were significantly correlated with the oral glucose tolerance test [10].

It is well known that diabetes has become a serious public health problem. Diabetes remains the fifth leading cause of death globally, although scientists have made great efforts to treat diabetes and prolong the life of patients. Compared with people without diabetes, the risk of premature death is increased by 15% in patients with type 1 and type 2 diabetes, and life expectancy is reduced by 10 and 20 years, respectively [11]. According to a systematic analysis, diabetes is the sixth leading cause of disability [12], and the multiple serious complications during the course of the disease bring huge economic pressure to patients and society. According to the International Diabetes Federation statistics in 2017, the number of global diabetics has reached 425 million, and it is estimated that by 2045, the number of diabetics in the world will reach 783 million [13]. Diabetes is mainly a metabolic disease characterized by high blood sugar caused by genetic factors, environmental factors, and unhealthy lifestyles, including type 1 diabetes, type 2 diabetes, and gestational diabetes mellitus. Among them, type 1 diabetes is caused by the immune-mediated destruction of pancreatic β cells and absolute insulin deficiency, which occurs mostly in adolescents. Type 2 diabetes is mainly caused by insulin resistance, and studies have found that Chinese patients with type 2 diabetes account for more than 90% of all diabetics, ranking first in the world and showing a rapid upward trend [14]. When hyperglycemia occurs during pregnancy, it is called gestational diabetes mellitus. It is estimated that in 2019, there were 20.4 million women with hyperglycemia during pregnancy, and the incidence of gestational diabetes mellitus in China was 14.8% [15]. In addition, more studies have found that environmental factors play an important role in the occurrence and development of diabetes [16,17], and new pollutants are also being considered [18].Therefore, the connection among exposure to emerging pollutants, glucose metabolism, and the development of diabetes is an area worthy of further exploration. However, the relationship between emerging pollutant exposure and glucose metabolism has yet to be systematically summarized and discussed.

Therefore, this review aims to explore the role of the gut microbiota in the toxic effects of emerging pollutants on glucose metabolism via systematically reviewing the existing literature. On the one hand, it offers new perspectives for understanding the hazards associated with emerging pollutant exposure, providing new research directions, data support, and scientific rationale for addressing the public health issue of diabetes. On the other hand, this review can provide evidence support for future research into the toxicity of emerging pollutants on glucose metabolism, drawing attention to the relationship between emerging pollutants and diabetes, and laying a foundation for the prevention and understanding of the toxicity of emerging pollutants.

## 2. Emerging Pollutants

**Figure 1:**
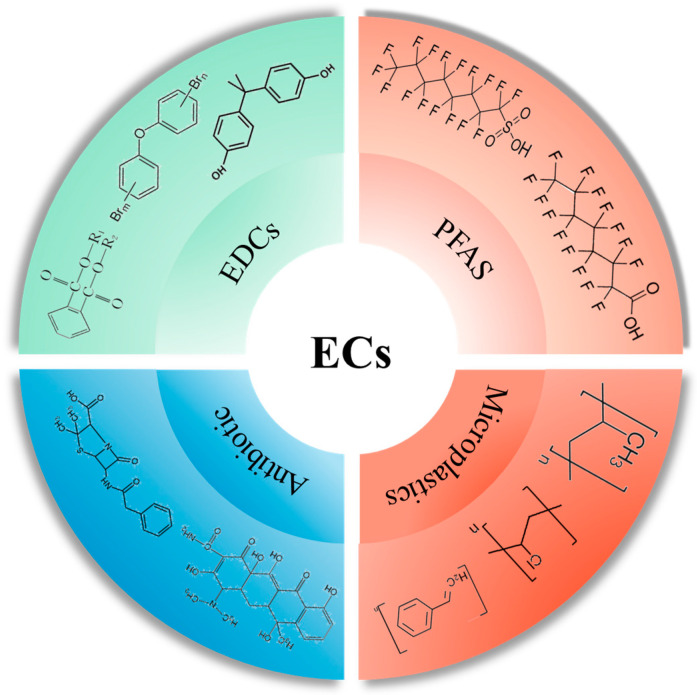
Typical emerging contaminants and representative compounds.

Emerging pollutants in the environment exhibit a broad spectrum and diverse origins. It is estimated that over 3000 different emerging pollutants have been detected in the environment [19]. The current literature categorizes these into four main groups: endocrine disruptors, perfluorinated compounds, microplastics, and antibiotics [20,21]. Compared to traditional environmental pollutants, emerging pollutants are characterized by (i) a degree of risk obscurity, due to their relatively recent identification and low environmental concentrations, making their short-term impacts less evident [22]; (ii) persistence in the environment, as they are difficult to metabolize and degrade and prone to bioaccumulation [23]; (iii) high toxicity to biological organisms, including humans, with many having endocrine-disrupting, carcinogenic, teratogenic, and mutagenic effects [24]; and (iv) challenges in management, owing to their vast variety and low concentrations, which complicates their detection and the understanding of their environmental and biological impacts [23,25]. The paucity of research on their environmental and biological harm, migration, and transformation mechanisms adds to the complexity of managing these pollutants. These characteristics have increasingly drawn attention to the need for in-depth research on emerging pollutants, providing a scientific foundation for their regulation and management (Figure 1).

### 2.1. Sources of Exposure to Emerging Pollutants

Scientists generally agree that dietary exposure is a major source of human exposure to emerging pollutants, with a wide range of pollution sources that cannot be ignored. Dietary pollutants mainly enter higher trophic levels through food chain transmission and nutritional transfer. For instance, studies on microplastics have shown that they are initially absorbed by plants (algae) in the environment, then ingested by consumers (like water fleas and freshwater fish), and ultimately ingested by apex consumers, including humans. It is shown that consuming 250 g of wet-weight mussels can result in an intake of approximately 90 particles of microplastics, and microplastics have been found to accumulate in human tissues such as the blood, placenta, and heart [26,27,28,29,30]. Wang et al. [31] detected 11 types of perfluorinated compounds in various consumer products like pork loin, pig heart, liver, kidney, chicken breast, and liver, with pig liver showing the highest average concentration of 3.438 ng/g, followed by pig kidney (0.508 ng/g). These findings indicate that emerging pollutants can enter and accumulate in biological organisms, including humans, through food chains and nutritional transfer, posing potential health hazards.

Studies measuring the concentration of polychlorinated biphenyls in Crassostrea tulipa (oysters) and Anadara senilis (mussels) found concentrations of 2.95–11.41 mg/kg and 5.55–6.37 mg/kg wet weight, respectively [32]. Apart from marine life, polychlorinated biphenyls and polybrominated diphenyl ethers have also been detected in commonly consumed milk samples [33], indicating potential endocrine-disrupting hazards from everyday dietary exposure. Studies on perfluorinated compounds found that younger populations have higher levels of contact with PFOS and PFOA, with dietary exposure primarily from fish, meat, eggs, and products containing these ingredients, with eggs and dairy products as major sources [34]. PFOS has been detected in meat, fish, shellfish, and fast food [35], and Ericson et al. estimated that an adult male’s dietary intake of PFOS could reach 62.5 ng/d [36]. In contrast, research on microplastics found that oral exposure is mainly concentrated in commercial fish, table salt, honey, and bottled water [37]. For instance, researchers found microplastics in various edible salts used by humans, with the highest concentrations in sea salt (550–681 particles/kg) [38]. Mason’s research also found that bottled water contains an average concentration of microplastics larger than 100 μm and smaller than 100 μm, at 10.4 particles/L and 325 particles/L, respectively. Cauwenberghe et al. also found microplastics in mussels, estimating that the maximum exposure to microplastics for adults through mussel consumption could reach 11,000 microplastics [28]. Antibiotics, long established in human food supplies, have gradually contaminated food products, including livestock, aquatic products, and vegetables [39]. Known for their use in treating and preventing diseases, as well as additives for promoting growth and improving feed efficiency, antibiotics are likely major contributors to contamination in livestock and aquatic products [40,41]. For example, amoxicillin and penicillin were detected in 81% and 27% of fresh milk samples, respectively [42]. Furthermore, an investigative study involving the random sampling of chicken and beef from supermarkets revealed a considerable detection rate of antibiotics. Quinolone drugs were identified in 45.7% of chicken samples and 57.7% of beef samples, with concentrations reaching 30.8 ± 0.45 μg/kg and 6.64 ± 1.11 μg/kg, respectively [43].

### 2.2. Connection between Emerging Pollutants and the Gut Microbiome

Contemporary research findings have demonstrated that exposure to emerging environmental pollutants alters the structure and composition of the gut microbiota. For instance, scientists, in a study examining the impact of nanoplastics on the gut microbiome, exposed Eriocheir Sinensis to polystyrene nanoplastics. Compared to the control group, the exposed group exhibited significant changes in the gut microbiota’s structure and composition, with a marked decrease in the relative abundance of Firmicutes and Bacteroidetes and an increase in Fusobacteria and Proteobacteria [44]. Another study found that mice exposed to 0.5 μm and 50 μm polystyrene microplastics showed significant alterations in the composition and structure of their gut microbiota, with a notable reduction in diversity and a decrease in the relative abundance of Firmicutes and α-Proteobacteria [45]. Additionally, research on PCB-126 revealed significant changes in the gut microbiota of exposed mice compared to the control group, with notable shifts in the proportion of Bacteroides, Parabacteroides, Romboutsia, and the ratio of Firmicutes to Bacteroidetes [46]. Recent studies in several species have found that Di-(2-ethylhexyl) phthalate (DEHP) exposure during development alters the structure and composition of the gut microbiota and reduces the diversity of the gut microbiota: an increase in the relative abundance of Firmicutes and Akkermansia was found, along with a decrease in the relative abundance of Bacteroidetes and Actinobacteria [47]. Furthermore, Yang et al. found that exposure to DEHP during infancy also altered the composition and diversity of the intestinal flora, with a decrease in the number of Rothia species and Bifidobacterium longum [48]. In their study of the effects of BPA on intestinal flora, Lai et al. found that the growth of TM7 and Proteobacteria was promoted in BPA-exposed mice, while the number of Clostridia was reduced [49]. Furthermore, studies have shown that gut bacteria such as Firmicutes and Bacteroides are significantly associated with type 2 diabetes [50].

## 3. Gut Microbiome, Diabetes and Potential Mechanisms

Diabetes, a metabolic disease characterized by abnormally elevated blood glucose levels, has become a significant global health issue due to its high prevalence and associated disability and mortality rates. In 2017, the estimated prevalence of diabetes in China was 12.8% [51], with the global diabetic population reaching 425 million people [52], over 90% of whom have type 2 diabetes [14], ranking it as the most prevalent and rapidly increasing trend worldwide. Genetic predisposition, environmental factors, and unhealthy lifestyles are closely linked to the onset and progression of diabetes. Moreover, recent studies have revealed an interesting phenomenon: the gut microbiome plays a crucial role in the development and progression of obesity and type 2 diabetes, with approximately 3.8 ± 0.2% of gut microbiota relative abundance associated with type 2 diabetes and obesity [50].

### 3.1. Diabetes and Gut Microbiota

In past diabetes research, the gut bacteria most commonly reported to be negatively associated with diabetes are Bifidobacterium and Bacteroides. The role of Bifidobacterium in type 2 diabetes appears to be consistently supported by the literature: Bifidobacterium potentially exerts a protective effect against type 2 diabetes [53,54]. For example, Gao et al. studied whether the composition and structure of the gut microbiota differed among healthy, overweight, and obese volunteers, finding a significant reduction in gut bacteria including Bifidobacterium, anti-inflammatory Faecalibacterium, and butyrate-producing Ruminococcaceae in the obese population compared to healthy individuals [55]. This phenomenon was validated in animal experiments: transplanting Bifidobacterium into mice on a high-fat diet, researchers found that Bifidobacterium not only reduced weight gain in mice but also significantly improved glucose–insulin disorder and hepatic steatosis, shifting the gut microbiota structure of high-fat diet mice towards that of normal-diet mice. Moreover, numerous cross-sectional studies have discovered a negative correlation between Bacteroides and type 2 diabetes. For instance, an analysis of the gut microbiota of 121 type 2 diabetes patients by Zhang et al. revealed dysbiosis and changes in alpha diversity, with a significant reduction in Bacteroides, only half the amount found in non-diabetic and pre-diabetic patients [56]. When mice were exposed to Bacteroides orally, researchers found that in high-fat-diet mice, not only were serum cholesterol, triglycerides, blood sugar, insulin, and leptin levels reduced, but their oral glucose tolerance was also improved [57].

Additionally, a few articles have reported gut bacteria positively correlated with diabetes or high blood sugar. Specifically, many studies have reported a positive correlation between Firmicutes, Ruminococcus, Lachnospiraceae, and Blautia with type 2 diabetes. For example, numerous population studies have indicated that both pre-diabetic and diabetic patients have relatively higher Operational Taxonomic Units (OTUs) of Ruminococcus. Furthermore, increases in the abundance of Sutterella, Streptococcus, Lachnospiraceae, Clostridiales, Eubacterium, Sporobacter, Abiotrophia, Firmicutes, and Subdoligranulum have been observed [58,59]. Moreover, type 2 diabetic patients have shown a notable decrease in the quantity of Ruminococcus and Lachnospiraceae following metformin treatment [60]. Some cross-sectional studies have found that compared with control groups, case groups have a marked increase in the quantity of Blautia, which diminishes after metformin treatment, thus affirming the significant role of Blautia in the development and progression of diabetes [56,61,62]. Additionally, researchers have discovered that compared with non-diabetic patients, the gut microbiome of diabetic patients is predominantly composed of opportunistic pathogens such as *Bacteroides caccae*, *Clostridium hathewayi*, *Clostridium ramosum*, *Clostridium symbiosum*, *Eggerthella lenta*, and *Escherichia coli* [50].

There is an evident link between emerging pollutants and abnormalities in glucose metabolism, with changes in the gut microbiota playing a significant role. However, it is important to note that not all studies on gut microbiota yield consistent conclusions. For instance, contrary to the studies mentioned above, Diamante, in his research on the impact of Bisphenol A on metabolic diseases, exposed pregnant mice to Bisphenol A and observed the metabolic phenotype of the offspring. The results indicated that male mice in the Bisphenol A exposure group had significantly lower insulin levels than those in the control group, and the area under the curve in the intraperitoneal glucose tolerance test was reduced. Diamante’s correlation analysis of differentially abundant amplicon sequence variants (ASVs) with the metabolic phenotype of mice found 18 ASVs related to body weight and two related to the area under the glucose tolerance curve [63]. Additionally, population studies have found an increase in the relative abundance of Firmicutes in both pre-diabetic and diabetic patients [59], whereas a decrease in Firmicutes was observed in high-fat-diet-induced obese mice [49]. The inconsistencies across different studies could be attributed to the use of varied animal models, which might affect metabolic levels and microbiota. Furthermore, the structure and composition of the gut microbiota are closely related to numerous factors, and studying the impact of just one exogenous chemical exposure is quite limited. In addition, the experimental conditions and environmental exposure during the experiment might affect the results and the composition of the gut microbiota.

### 3.2. Potential Mechanisms of Gut Microbiota-Induced Glucose Metabolic Abnormalities

**Figure 2:**
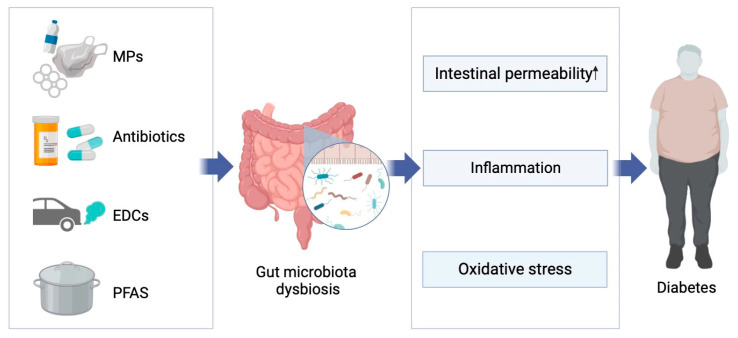
Possible mechanisms of exposure to emerging pollutants affecting diabetes through gut microbiota dysbiosis.

The gut microbiota impacts host metabolism through various pathways, including inflammatory responses, intestinal permeability, glucose metabolism, and the collective action of the microbiome. It is well established that certain gut bacteria and their metabolites can alter levels of pro-inflammatory and anti-inflammatory factors, as well as lipopolysaccharides (LPS), in the host. Given that inflammation and inflammatory mediators are closely linked with the development of type 2 diabetes, the influence of the gut microbiota on glucose metabolism through inflammatory responses has garnered widespread attention. For example, several studies have reported that patients with type 2 diabetes exhibit elevated serum endotoxin levels, a decrease in butyrate-producing bacteria and Firmicutes abundance, and an increase in Lactobacillus and Betaproteobacteria abundance. These findings suggest that certain gut bacteria may induce type 2 diabetes through endotoxin-induced inflammatory responses [50,64,65]. Chen et al., in their research on the role of Lactobacillus in diabetes progression, found that, besides significantly reduced fasting blood glucose and postprandial 2 h blood glucose levels, exposed mice showed decreased levels of pro-inflammatory cytokines such as TNF-α and LPS and increased levels of anti-inflammatory cytokines like IL-10 [66]. Another critical feature of type 2 diabetes is increased intestinal permeability, which allows gut microbiota and their metabolites to enter the bloodstream. Chelakkot et al. [67] found that, compared to diabetic patients, healthy individuals had a higher number of extracellular vesicles from *Akkermansia muciniphila* (AmEVs) in their fecal samples, which also enhanced the function of tight junction proteins in the intestines of diabetic mice. To verify their direct effect, Chelakkot applied AmEVs to lipopolysaccharide-treated Caco-2 cells and observed improved cellular permeability and increased the expression of the occludin protein. These findings suggest that AmEVs can regulate the expression of key proteins and affect intestinal permeability functions. Importantly, the gut microbiota might also alter blood glucose levels by affecting glucose homeostasis and insulin resistance in primary metabolic organs like the liver, muscles, and adipose tissue. Dang et al. found that after treating diabetic mice with *Lactobacillus paracasei*, not only were the mice’s fasting blood glucose, postprandial blood glucose, and glucose tolerance adjusted, but the expression of genes associated with gluconeogenesis, such as G-6-Pase and PEPCK, was inhibited, and levels of IRS-2, PI3K, and Akt were increased to normal. Additionally, the treatment of diabetic mice with L. casei CCFM419 effectively improved the downregulated mRNA expression levels of PI3K and GS and significantly decreased the expression of the GSK3β gene [68]. Besides these potential mechanisms, some gut bacteria might also affect host physiology through interactions with other bacteria [69,70] (Figure 2).

## 4. Emerging Pollutants, Gut Microbiome, and Diabetes

Given the known role of the gut microbiome in the development and progression of diabetes, coupled with the association between emerging pollutant exposure and changes in the gut microbiome, it is plausible that the disruption of glucose metabolism via the gut microbiome may be a potential mechanism by which emerging pollutants contribute to diabetes. This section will discuss, category by category—microplastics, antibiotics, endocrine disruptors, and perfluorinated compounds—their impacts on glucose metabolism and the role and function of the gut microbiome therein.

### 4.1. Microplastics

The concept of microplastics, defined as plastic particles with a diameter of ≤5 mm, was formally introduced by Thompson et al. in 2004 [71]. Since then, scientific research on microplastics present in the environment has become increasingly extensive. Numerous studies have found that plastics in the environment, upon degradation through physical, chemical, and biological processes into microplastic particles, pose toxicological threats to the natural environment, ecosystems, and flora and fauna [15,72,73,74]. Many studies focus on the significant impacts of microplastics entering the human body through various pathways on glucose metabolism and their potential mechanisms.

In recent years, more scientists have begun to focus on changes in the gut microbiota of diabetic mice following microplastic exposure, attempting to elucidate the relationship between the toxicity of microplastics to the gut microbiota and sugar–lipid metabolism (Table 1). For instance, Shi et al. [75] exposed mice to 1 μm polystyrene microplastics and found an increase in fasting blood glucose and insulin levels, hypothesizing that this may be due to a disruption of the gut–liver axis prompted by changes in the structure and composition of the gut microbiota. An analysis of the gut microbiome revealed a marked reduction in its diversity and significant changes at the phylum level, with a decrease in the abundance of Bacteroidetes and Verrucomicrobia and an increase in Firmicutes, Deferribacteres, and Actinobacteria. Similarly, another study arrived at consistent results, with a decreased abundance of Bacteroidetes in mice fed a high-fat diet containing microplastics, also noting reductions in the Chao1, Shannon, and Gini–Simpson indices [76]. Huang et al. [77] also found that after microplastic exposure in high-fat-diet mice, there was a reduction in microbial richness and diversity, with a relative increase in the abundance of Gram-negative rods (such as Prevotellaceae and Enterobacteriaceae). However, intriguingly, a different conclusion was reached in another study. Liu et al. [78] examined the varying responses of healthy mice and diabetic mice to exposure to polystyrene microplastics, finding that the gut microbiota in both healthy and diabetic mice changed post exposure. The difference was that in healthy mice, the proportion of probiotics (Alloprevotella, Bacteroides, Dubosiella, Lachnospiraceae_NK4A136_group, Lactobacillus, Weissella) declined while the proportion of pathogens (Helicobacter, Parabacteroides, Candidatus_Saccharimonas, Lachnoclostridium) increased; in contrast, diabetic mice exhibited an increased proportion of probiotics and a decreased proportion of pathogens.

### 4.2. Antibiotics

Developed in the late 1940s for treating bacterial pathogen-induced infections, antibiotics are known to selectively target potential pathogens within microbial populations while significantly disrupting the human gut microbiome, with effects lasting for several months or even longer [79,80]. Recent studies have begun to explore the impact of antibiotic treatment on the gut microbiome and human metabolism.

Vrieze et al. [81] conducted a study to investigate the effect of vancomycin on insulin sensitivity and the gut microbiota. Patients with metabolic syndrome were randomly assigned to different treatment groups. They found that after one week of treatment, patients in the vancomycin group showed a significant decrease in insulin sensitivity and noticeable changes in fecal microbiome diversity, including a reduction in the relative abundance of Gram-positive bacteria and a compensatory increase in Gram-negative bacteria. These results highlight the influential role of the gut microbiome in how antibiotics affect glucose metabolism. Additionally, Hwang’s animal study showed that antibiotics alter the structure and composition of the gut microbiome, enhancing the microbes’ capacity to collect and store energy, thereby changing insulin sensitivity and glucose tolerance in mice [82,83]. In addition, in an experiment that treated ob/ob mice with Ampicillin and neomycin, it was found that compared with mice that did not receive antibiotic treatment, the treated mice not only showed better glucose tolerance but also showed significant changes in the structure and composition of their gut microbiota, which was only 22% similar to that of the mice on the high-fat diet before the treatment [84]. Another study found that antibiotic therapy improved fasting glucose, glucose tolerance, and gut microbiota in mice [85]. It is well known that gut bacteria ferment carbohydrates in the intestine into short-chain fatty acids [86], which, in turn, affect metabolism and energy balance by altering the expression and secretion of intestinal hormones. For instance, Livanos used antibiotics to treat mice, finding a reduction in the diversity of the gut microbiome and the selection of unique microbial community structures and a significant increase in diabetes incidence [87].

### 4.3. Endocrine Disruptors

Beyond the aforementioned microplastics and antibiotics, endocrine disruptors represent another significant category of emerging pollutants in the environment, one that is gradually garnering scientific attention. Environmental endocrine disruptors primarily include phenolic compounds, pesticides, and persistent organic pollutants. These disruptors enter organisms through various exposure pathways and gradually accumulate in tissues and organs [88,89,90], interfering with normal hormone synthesis and secretion, leading to hormonal imbalances and consequent endocrine diseases like obesity and diabetes [91].

Yan et al. studied endosulfan sulfate (ES) exposure in pregnant mice and found that ES inhibits high-fat-diet-induced adipogenesis, reduces glucose tolerance, and affects glucose homeostasis by promoting lipolytic metabolism and fatty acid oxidation and altering the composition of the intestinal flora [92]. Meanwhile, Fan et al. showed that pregnant mice exposed to DEHP had offspring with abnormalities in adipogenesis, energy expenditure, glucose tolerance, and dysbiosis of the intestinal flora, and linear discriminant analysis (LDA) showed significant differences in 16 characteristic flora at the phylum and genus levels between exposed and control mice. Meanwhile, Fan et al. showed that the offspring of mice exposed to DEHP during gestation had abnormal lipogenesis, energy expenditure, and glucose tolerance and had dysbiosis in the gut microbiota. LDA analysis showed that the 16 characteristic flora of exposed mice and control mice were significantly different at the phylum and genus levels [93]. However, relatively few studies have been conducted on the disruption of glucose metabolism by affecting the structure and composition of the intestinal flora after exposure to EDCs. Nevertheless, numerous studies have found that exposure to endocrine disruptors affects glucose metabolism, leading to elevated blood glucose levels. Marmugi et al. exposed CD-1 mice to bisphenol A and observed that compared to the control group, the exposed mice had significantly higher blood glucose and plasma cholesterol levels. Moreover, mice exposed to a dose of 5000 mg/kg/d showed decreased glucose tolerance and a significant increase in the area under the curve [94]. Many studies have drawn similar conclusions, finding that C57BL/6 mice exposed to bisphenol A, even on a normal diet, showed an increase in body weight, elevated insulin levels, and impaired glucose tolerance, with bisphenol A exacerbating high-fat-diet-induced weight gain and insulin resistance in mice [95,96]. Interestingly, Lai et al. exposed CD-1 mice to bisphenol A and found that it induced gut microbiome community structures similar to those induced by a high-fat diet, with an increased relative abundance of Proteobacteria and Helicobacteraceae and a decrease in Firmicutes and Clostridiapopulations [49]. Consequently, the role of the gut microbiome in endocrine disruptor-induced glucose metabolic disorders is increasingly recognized. Tian et al. [46] exposed C57BL/6 high-fat-diet mice to polychlorinated biphenyl-126 and found that early-life exposure to PCB-126 in mice led to decreased glucose tolerance and a significant increase in metabolites involved in the tricarboxylic acid cycle (e.g., pyruvate, succinate, citrate). Tian concluded from these results that early exposure to PCB-126 exacerbates glucose homeostasis impairment characterized by abnormal glucose tolerance and increased tricarboxylic acid cycle flux in high-fat-diet mice. Additionally, researchers have found that PCB-126 affects the structure and composition of the mouse gut microbiome. For example, compared to control mice, high-fat-diet mice exposed to PCB-126 in early life showed a significant decrease in the relative abundance of Muribaculum, Duncaniella, Bacteroides, Parabacteroides, and Prevotella in the cecum, while the ratio of Firmicutes/Bacteroidetes, Romboutsia, and Adlercreutzia significantly increased. Qin et al.’s study found that the aforementioned groups, such as Firmicutes, Clostridium, Bacteroides, and Parabacteroides, are significantly associated with type 2 diabetes [50]. Li et al.’s research on the impact of TCDD exposure during pregnancy and lactation in mice found significant changes in the structure and composition of the gut microbiome, characterized by an upregulation of Firmicutes, Bacteroidetes, Clostridia, and Lachnospiraceae. Moreover, Pearson correlation coefficients suggested that affected tryptophan metabolism (positively correlated with type 2 diabetes) was positively correlated with harmful bacteria and negatively correlated with beneficial bacteria [97,98].

### 4.4. Perfluorinated Compounds

Similar to endocrine disruptors, perfluorinated compounds such as Perfluorooctane sulfonate (PFOS) and Perfluorooctanoic acid (PFOA) are characterized by unique structures and stability, allowing them to persist in the environment and accumulate in organisms following exposure, thereby posing health risks [99,100,101,102,103].

Perfluorinated compounds are also ubiquitously present in the environment and pose significant threats to the organisms living in it, particularly regarding metabolic effects. Consequently, scientists are focusing on the impact of perfluorinated compound exposure on glucose metabolism by disrupting flora metabolism. Wei et al. exposed adult male mice to 25 mg/kg/d DEHP (Di-(2-ethylhexyl) phthalate) via continuous oral exposure, finding that the mice developed elevated fasting blood glucose levels and hepatic fat accumulation. Interestingly, research on the intestinal flora of mice revealed significant differences in the community structure of the gut microbiota of exposed mice compared to the control group. Moreover, LDA showed that 29 features were significantly different between control and exposed mice from the gate level to the genus level. Compared to control mice, the relative abundance of cyanobacteria in the intestinal flora of exposed mice was significantly increased at the phylum level, whereas at the genus level, the relative abundance of Bacteroides was decreased, and the relative abundance of Allobaculumin was increased [104]. However, relatively few studies have been conducted in this area, and current articles have focused on the effects on glucose metabolism following exposure to PFAS or changes in gut microbiota caused by exposure to PFAS. Rats exposed to PFOS during gestation exhibited pre-diabetic symptoms in their offspring, with elevated fasting insulin and leptin levels and impaired glucose tolerance compared to the control group. Lv inferred from these results that exposure to PFOS during development could lead to glucose metabolism disorders in adulthood in rats [105]. Yan’s study arrived at a similar conclusion: mice exposed to PFOA showed higher insulin sensitivity and glucose tolerance and reduced hepatic glycogen synthesis compared to the control group [106]. Additionally, researchers found that mice exposed to PFOS exhibited disorders in fat and glucose metabolism [107]. Interestingly, upon analyzing the gut microbiota of mice, researchers found a significant increase in the relative abundance of Turicibacterales and Turicibacteraceae in the exposed group; the glucose metabolism disorder in mice was notably positively correlated with the relative abundance of Turicibacteraceae [61,107]. Furthermore, an increase in the relative abundance of Allobaculum, which contributes to insulin resistance and obesity in mice, was found in the exposed mice. Significant changes in the relative abundance of Turicibacter, Allobaculum, B. acidifaciens, and Dehalbacteriaceae, which are considered related to dysregulation of sugar and lipid metabolism, were observed [107]. In studies on OBS (Sodium ρ-perfluorous nonenoxybenzene sulfonate, a PFASs substitute), it was found that OBS exposure in zebrafish led to a decrease in cytoplasmic phosphoenolpyruvate carboxykinase gene levels in the liver (related to glucose metabolism levels), with a decrease in the relative abundance of β-Proteobacteria, Bacteroidetes and Actinobacteria, α-Proteobacteria, γ-Proteobacteria, and Verrucomicrobia [108]. Moreover, exposure to F-53B (a PFOS substitute) also caused an increase in the relative abundance of Verrucomicrobia and a decrease in Firmicutes in the gut microbiome of mice, with significant changes in Akkermansia, Bacteroides, and Ruminococcus (significantly related to type 2 diabetes) [50,109].

However, unlike microplastics and antibiotics, few studies on EDCs and PFAS have addressed the effects of gut microbiota disruption on glucose metabolism. Nevertheless, we reviewed the effects of EDCs and PFAS on the gut microbiota and analyzed the possible correlation between altered gut microbiota and glucose metabolism (Table 2).

## 5. Conclusions and Outlook

In recent years, more studies have begun to focus on the impact of exposure to emerging pollutants in the environment on metabolic disorders, particularly glucose metabolism disorders. Given the significant role of the gut microbiota in glucose metabolism, scientists are starting to pay attention to the structural and compositional disorders of the gut microbiota caused by pollutants, as well as changes in microbial diversity. Although an increasing number of studies are focusing on the role of the gut microbiota in glucose metabolism disorders caused by emerging pollutants, current research is just the tip of the iceberg, and our understanding in this area remains very limited:

(1) Many studies have shown that exposure to emerging pollutants can cause pre-diabetic symptoms or exacerbate existing glucose metabolism disorders in organisms. Interestingly, different results have been found for the same compound, possibly due to different diabetes animal models or exposure periods used in the studies. Therefore, there is an urgent need for more in-depth research to standardize diabetes animal models or exposure forms.
(2) It is well known that the gut microbiota plays a crucial role in the development of diabetes. However, studies on whether exposure to emerging pollutants affects the glucose metabolism process by altering the structure and composition of the gut microbiota are relatively scarce. Additionally, there is still controversy over changes in certain specific gut bacteria like Akkermansia, Parabacteroides, and Verrucomicrobia after exposure to emerging pollutants or during glucose metabolism disorders. Therefore, more research is needed to explore the changes in the gut microbiota after exposure to emerging pollutants and its relationship with glucose metabolism.
(3) Most current research on the impact of emerging pollutants on the gut microbiota and glucose metabolism focuses on animal models. Due to interspecies differences, studies on the impact of emerging pollutants on human populations are very limited. Therefore, large-scale population studies are needed to elucidate the impact of emerging pollutant exposure on human glucose metabolism and the role of the gut microbiota in this process.
(4) Currently, most research on emerging contaminants focuses on the effects of exposure on glucose metabolism or on a single aspect of the gut microbiota, while relatively few studies have been conducted on whether they affect the development of diabetes by altering biological glucose metabolism through the gut microbiota, especially with regard to endocrine disruptors and perfluorinated compounds. Therefore, large-scale and more in-depth studies are needed to elucidate whether exposure to emerging contaminants causes glucose metabolism disorders through the gut microbiota and its specific mechanisms.

## Figures and Tables

**Table 1:** Effects of microplastic exposure on glucose metabolism and gut microbiota.

Species	Microplastics	Altered Glucose Metabolism	Altered Gut Microbiota	Reference
ICR mice	Polystyrene microplastics (1 μm)	Fasting blood glucose, fasting insulin, and HOMA-IR levels were significantly elevated in mice after microplastics exposure.	pCoA analysis showed that the diversity of the gut microbiota was significantly reduced in the exposure group mice compared to the control mice. At the phylum level, the relative abundance of Bacteroidetes and Verrucomicrobia was significantly reduced in the exposure group mice, whereas the relative abundance of Firmicute, Deferribacteres, and Actinobacteria was significantly increased. At the genus level, the relative abundance of Oscillospira, Akkermansia, and Desulfovibrio was decreased, and the relative abundance of Lactobacillus and Bifidobacterium was increased in the exposure group mice. Further analysis showed that the relative abundance of Bacillales, Staphylococcaceae, Jeotgalicoccus, and Rikenella was significantly altered in the exposure group mice.	[75]
High-fat-diet mice (C57BL/6J)	Polystyrene microplastics	In the IPGTT (intraperitoneal glucose tolerance test), ND and HFD mice fed added microplastics had higher blood glucose levels than both ND and HFD mice. In the ITT (insulin tolerance test), HFD mice with added microplastics had higher blood glucose levels than HFD mice.	At the gate level, the relative abundance of Bacteroidetes was decreased, and the relative abundance of Proteobactria was increased in the exposure group mice. The Chao 1 index, Shannon index, and GiniSimpson index of gut microbiota abundance decreased in the exposure group mice.	[76]
ICR mice	Polystyrene microplastics (5 μm, 50 μm, 100 μm, 200 μm)	Fasting glucose and fasting insulin levels were significantly elevated in mice, and the AUC of OGTT and ITT was elevated.	Significant changes in the structure and composition of the gut microbiota. At the phylum level, the relative abundance of Bacteroidetes was elevated, and the relative abundance of Firmicutes decreased, causing a decrease in the Firmicutes-to-Bacteroidetes ratio. At the family level, the relative abundance of Muribaculaceae and Helicobacteraceae decreased, and the relative abundance of Prevotellaceae, Enterobacteriaceae, Desulfovibrionaceae, and Rikenellaceae increased. The relative abundance of Prevotellaceae and Enterobacteriaceae increased.	[77]
db/db mice	Polystyrene microplastics (100 nm)	Elevated fasting blood glucose levels in mice after microplastic exposure suggested impaired glucose control. Mice in the exposure group had significantly higher blood glucose levels at 90 min and 120 min after oral glucose administration in the OGTT test.	Microplastic exposure increased gut microbiota diversity in healthy mice and decreased gut microbiota diversity in diabetic mice. Microplastic exposure resulted in a decrease in the proportion of probiotic bacteria and an increase in the proportion of pathogenic bacteria in healthy mice gut microbiota, whereas diabetic mice gut microbiota showed an increase in the proportion of probiotic bacteria and a decrease in the proportion of pathogenic bacteria.	[78]

**Table 2:** Effects of EDCs and PFAS on glucose metabolism and gut microbiota.

Species	Chemical	Altered Gut Microbiota	Gut Microbiota Associated with Glucose Metabolism	Reference
Pregnant CD-1 mice	Endosulfan sulfate	Alleviation of obesity and liver triglyceride accumulation due to high-fat diet. Elevated fasting blood glucose and reduced glucose tolerance.	There was a reduced α diversity of gut microbiota in the exposure group of mice. ES treatment alleviated high-fat-diet-induced increases in the relative abundance of Actinobacteria and Proteobacteria. ES treatment alleviated high-fat-diet-induced increases in the relative abundance of Enterorhabdus and Bifidobacterium. Chronic exposure to ES caused an increase in the relative abundance of Bacteroides.	[92]
Pregnant mice	Di-(2-ethylhexyl)-phthalate	Reduced glucose tolerance and disturbed glucose metabolism in offspring mice. Lifelong metabolic consequences for offspring in a gender-dependent manner.	LDA analysis showed that mice in the offspring of the exposed group were significantly different at the gate level for 16 features. There was an increased α diversity of gut microbiota in offspring mice of the exposure group.	[93]
CD-1 mice	Bisphenol A	Compared with the control mice, the exposure mice showed a significant reduction in the diversity of the gut flora. α-diversity and β-diversity analyses suggest that BPA leads to a gut microbiota community structure similar to that induced by high-fat diets. Proteobacteria were significantly elevated in both bisphenol A exposure group mice and high-fat dietary mice.	Elevated Helicobacteraceae and Proteobacteria and reduced Firmicutes and Clostridiapopulations were observed in both BPA-exposed mice and high-fat-diet mice.	[49]
High-fat-diet mice	Polychlorinated Biphenyl 126	High-fat-diet mice exposed to PCB126 early in life showed a significant decrease in the relative abundance of Muribaculum, Duncaniella, Bacteroides, Parabacteroides, and Prevotella and an increase in the relative abundance of Romboutsia, Akkermansia, and Adlercreutzia.	Early exposure to PCB126 significantly decreased the abundance of Bacteroidetes and increased the ratio of Firmicutes/Bacteroidetes in mice on a high-fat diet, whereas the ratio of Firmicutes/Bacteroidetes was not significantly altered, and the relative abundance of Firmicutes and Verrucomicrobia was significantly altered in mice on a normal diet.	[46]
C57BL/6 mice (pregnant and lactating mice)	2,3,7,8-tetrachlorodibenzo-p-dioxin	Alterations in the structure and composition of the gut microbiota of both mothers and offspring, reflected in an upregulation of harmful bacteria and a downregulation of beneficial bacteria.	The dominant phylum in mothers and offspring of mice in the exposure group was Firmicutes and Bacteroidetes; the dominant class was Bacteroidia and Clostridia; the dominant order was Bacteroidales and Clostridiales; and the dominant families were S24-7 and Lachnospiraceae.	[97]
CD-1 mice	Di-(2-ethylhexyl) phthalate	Significant increase in blood glucose levels and hepatic fat accumulation in mice in the exposed group.	Significant reduction in the alpha diversity of the gut microbiota of mice in the exposure group. Significant increase in Cyanobacteria relative abundance at the gate level. Significant increase in the relative abundance of Allobaculumin and a decrease in the relative abundance of Bacteroides at the genus level.	[104]
CD-1 mice	Perfluorooctane sulfonic acid	Metabolic disorders, especially fat and glucose metabolism, occurred in exposure group mice. The relative abundance of Firmicutes, Bacteroidetes, Proteobacteria and Cyanobacteria were altered.	Disturbed glucose metabolism was positively associated with Turicibacteraceae, similar to the increase in Turicibacterales in previously hypercholesterolemic fed mice. An increased abundance of Allobaculum (a hypothesized short-chain fatty acid-producing bacterium) in PFOS-exposed mice contributed to insulin resistance and obesity. There were significant changes in the abundance of Turicibacter, Allobaculum, B. acidifaciens, and Dehalbacteriaceae, which are thought to be associated with disturbed glycolipid metabolism.	[107]

## Data Availability

Not applicable.
